# COVID-19-Related Psychological Trauma and Psychological Distress Among Community-Dwelling Psychiatric Patients: People Struck by Depression and Sleep Disorders Endure the Greatest Burden

**DOI:** 10.3389/fpubh.2021.799812

**Published:** 2022-01-07

**Authors:** Amira M. Ali, Abdulmajeed A. Alkhamees, Eman S. Abd Elhay, Samah M. Taha, Amin O. Hendawy

**Affiliations:** ^1^Department of Behavioral Medicine, National Institute of Mental Health, National Center of Neurology and Psychiatry, Tokyo, Japan; ^2^Department of Psychiatric Nursing and Mental Health, Faculty of Nursing, Alexandria University, Alexandria, Egypt; ^3^Department of Medicine, College of Medicine and Medical Sciences, Qassim University, Buraydah, Saudi Arabia; ^4^Department of Psychiatric Nursing and Mental Health, Faculty of Nursing, Mansoura University, Mansoura, Egypt; ^5^Department of Biological Production, Tokyo University of Agriculture and Technology, Tokyo, Japan; ^6^Department of Animal and Poultry Production, Faculty of Agriculture, Damanhour University, Damanhour, Egypt

**Keywords:** coronavirus disease 2019/COVID-19, psychological trauma, psychological distress, psychiatric disorders/co-morbid physical disorders, stay-at-home, major depression disorder/sleep disorders, age/unemployment/single/marital status, Arabic/Arab/Saudi Arabia

## Abstract

COVID-19 has created a general state of worry and distress, especially among vulnerable groups such as those with psychiatric diagnoses. Worldwide, psychiatric care provision has drastically suffered during the pandemic, with many patients unable to access proper care, which may have implications for increased mental health consequences in patients with psychiatric disorders (e.g., relapse and suicide). This cross-sectional study used structural equation modeling to investigate COVID-19-related trauma and distress among Arab psychiatric population during COVID-19 quarantine. Patients with pre-existing psychiatric disorders (*N* = 168) completed an online survey that comprised the Depression Anxiety Stress Scale 21 (DASS-21), the Impact of Event Scale-Revised (IES-R), and a questionnaire on COVID-19-related attitudes/perceptions, sources of information, used protective measures, and socio-demographic information. Respondents commonly reported feeling down-hearted/blue, trouble concentrating, along with symptoms of avoidance and rumination related to the pandemic. Patients with depression and sleep disorders expressed higher COVID-19-related trauma than patients with other disorders. Perceived physical health mediated the effect of co-morbid chronic physical disorders on COVID-19 trauma, psychological distress, perceived vulnerability to COVID-19, and perceived likelihood of recovery in case of contracting COVID-19. Perceived physical health and perceived vulnerability to COVID-19 were strong direct predictors of COVID-19-related trauma and psychological distress. Staying at home negatively predicted COVID-19 trauma and exerted an indirect negative effect on psychological distress via COVID-19 trauma. COVID-19 trauma, age, and marital status directly predicted psychological distress, with COVID-19 trauma being the strongest predictor. Educational level, income, having family members working in the medical field, keeping up to date with the news on deaths/infected cases or the development of COVID-19 drugs or vaccines, satisfaction with available information on COVID-19, and using different protective measures were not associated with significant differences in COVID-19 trauma and psychological distress scores. Immuno-psychiatric interventions should be designed to target COVID-19-trauma and distress among younger single patients with perceived poor physical health, especially those diagnosed with depression and sleep disorders.

## Introduction

The ongoing coronavirus disease 2019 (COVID-19) pandemic has been associated with the flaring of numerous psychological symptoms such as fear, anxiety, depression, stress, worry, anger, traumatic emotional experiences, and hopelessness in the general public since it first erupted in 2019 until now (1). Among 140732 individuals across 103 studies conducted during the COVID-19 outbreak, the prevalence of anxiety was 27.3% (95% CI: 23.7 to 31.2%) in the general population and 39.6% (95% CI: 30.1 to 50.1%) in COVID-19 patients (2). The levels of distress and trauma symptoms develop at higher levels in individuals who have been in contact with COVID-19 patients (e.g., healthcare providers and family members of COVID-19 patients) due to the development of vicarious trauma (3, 4). However, the general public and vulnerable groups are not exempted from experiencing negative emotional reactions. This is because of numerous distressing features of the pandemic: (1) wide geographical expansion of the disease, (2) announcement of COVID-19 as a global pandemic by the World Health Organization (WHO) entailing confirmed information on human-to-human transmission of the disease, (3) aggressive nature of the disease and rising death rates, (4) lack of disease-specific treatments, (5) uncertainty concerning the protective effects of evolving vaccines, (6) economic consequences of the outbreak, and (7) terrorizing images and stories of the pandemic communicated by mass media and social media (1, 5–10).

In several instances, stories informed about COVID-19 involve propagated and dangerously inaccurate beliefs, which support the contagion of fear alongside the disease itself (11–15). In particular, fears frequently reported are relevant to the negative impact of the pandemic on household finances of individuals and their significant others, unavailability of health care, insufficient food supply, job loss/unavailability, and excessive fear of contracting the disease (1, 6, 16, 17). In fact, Arpaci and colleagues have developed a measure of COVID-19 phobia based on criteria described in disease classification systems such as DSM-IV (8). In accordance, several studies reported negative consequences of COVID-19 phobia in different parts of the world (6–8, 17). Death due to lack of presenting to the hospital because of fear of contracting COVID-19 is a documented example (17).

Social distancing, primarily being locked down at home has been adopted in most countries as the most protective strategy against COVID-19. However, this strategy may cause several negative physical and psychological problems such as obesity, depression and domestic violence (18–20). For large-size families, especially with children under the age of 18 years, prolonged exposure to human sounds within the context of home confinement may cause excessive sensory input, sense of crowding—especially in small-size households, and lack of privacy leading to detrimental effects on health and well-being (21, 22). Large-scale studies show that being in self-isolation during COVID-19 was associated with greater depression, health anxiety, generalized anxiety disorder (GAD), financial worry, insomnia, acute stress, and loneliness among adults in the United States (US) (23, 24). The number of days in isolation correlates with the intensity of COVID-19-related distress (25). Meanwhile COVID-19 fear, deficient coping, and vicarious trauma associated with frequent exposure to social media/news concerning COVID-19 are identified mechanisms for increased COVID-19 psychopathology during the lockdown, especially in psychiatric/neurological patients, women, young age, and students (26, 27).

Imposed isolation, along with false or misleading information about COVID-19, may tigger a sense of perceived loss of control and jeopardize people's existential need to feel safe. Fuelled by alarmist saturation publicity, conspiracy theories—illogical, erroneous, and unhelpful disease-related beliefs/arguments (e.g., the virus causing COVID-19 is man-made)—propagate (28–30). COVID-19 associated conspiracy beliefs spread in a manner analogous to a virus (15, 29). Conspiracy beliefs develop stronger in response to widespread and significant events, which are enclosed within contradiction, uncertainty, misinformation, or unsatisfactory mundane explanations. These beliefs are largely endorsed by distressed individuals to help them achieve a sense of comfort. They operate by promoting cognitive closure—lower attention to and misappraisals of anomalous/threatening stimuli, increasing the occurrence of perceptual abnormalities and persecutory ideation (11, 12, 28, 31). An investigation involving community-dwelling individuals in the UK early during the pandemic reports that COVID-19 news moderated the effect of low political trust and COVID-19 fear on psychotic-like experiences (e.g., paranoia, hallucinations, and compulsive buying), especially among employees and students (32). Meanwhile, hospitalized psychiatric patients expressed a belief that the hospital staff orchestrated the pandemic to restrict leave and delay discharge (28). Indeed, psychiatric patients demonstrate increased proneness to COVID-19 conspiracy beliefs (28, 33), which are evoked by several liability factors including environmental conditions and psychological processes: low socioeconomic status (e.g., being unmarried and low level of education), powerlessness, perceptions of alienation from decision makers and breakdown in containment and social order, increased health-related concerns, adverse childhood experiences, maladaptive personality traits such as schizotypal and paranoia, psychiatric problems, as well as other non-psychotic psychological characteristics (e.g., social isolation, stress) (28, 31).

A longitudinal study evaluated the emotional impact of COVID-19 (posttraumatic stress as well as depression, anxiety, and stress symptomatology) in the general public in China twice over the course of 4 weeks. It reported reduction in the intensity of COVID-19-related traumatic stress over time. However, the intensity of trauma was significantly above the cut-off point at both instances. Meanwhile, the intensity of the symptoms of depression, anxiety, and stress was significantly high at both measurements (34). A meta-analysis of longitudinal studies reports a slight significant increase in mental symptomatology early during the pandemic. However, symptoms of anxiety and general mental dysfunction declined by mid-2020 while the levels of depression remained persistently high (35). Thus, adaptation to the prolonged pandemic may lessen the trauma but does not abolish it and associated symptoms of emotional negativity (34, 35). Likewise, the feeling of loneliness during strict lockdown is reported to decrease over time among the general public. However, some individuals (e.g., unemployed and unmarried) may still experience intense loneliness (36). Various social factors are reported to interfere with psychological responses and resilience during the pandemic (37). For example, psychological distress is higher among individuals with female gender, student status, young age, single social status, employment, increased number of people in the household (3–5 persons), change in daily routine, and loss of income (25, 26).

People vulnerable to stress, who usually have low social support, coping problems, and poor adaptation, may develop psychopathology and severely suffer under conditions of collective distress such as the current crisis of the global COVID-19 pandemic (19, 38–41). COVID-19 phobia is reported to increase depression, anxiety, phobic-anxiety, paranoia, obsession-compulsion symptoms, emotional coping, and dysfunctional behaviors in the general population (26, 32, 42). Meta-analytic data emphasize that pre-existing psychiatric illnesses represent a key risk factor for increased mental distress during COVID-19 (27). Available data show worsening in the levels of psychiatric symptoms such as anxiety, depression, stress, insomnia, suicidal ideation, impulsivity, posttraumatic stress symptoms, and dysfunctional eating in patients with pre-existing psychiatric disorders during the COVID-19 pandemic (41, 43, 44). Indeed, COVID-19 related fear/anxiety is reported to trigger relapse in a remitting patient with schizophrenia (38) as well as in two elders with depressive disorder (45). Apart from those case studies, an investigation during COVID-19 lockdown in India reports relapse in 30% of 132 patients with severe mental disorders who were stable before COVID-19. Stopping psychiatric medications was evident in one out of five patients, and it was associated with worsening of psychiatric symptoms (46).

Challenges regarding limiting COVID-19 transmission among psychiatric inpatients and caregivers have drastically affected the provision of psychiatric care across the world during the COVID-19 crisis. There is more dependence on telemedicine (telepsychiatry, even at the emergency department), restrictions on hospital admission, and enrolling patients into COVID-19 positive and negative units based on testing for COVID-19 status (47, 48). Although the use of telepsychiatry has increased in many Arab countries after COVID-19, several barriers (relevant to patients and systems) render this service less effective for counseling and treatment (49). In the meantime, some small- to medium-sized psychiatric hospitals also refuse to receive new inpatients because of poor medical conditions, which would possibly deteriorate distress symptoms for patients with mental illness (47).

In addition to being unable to access proper healthcare, the pandemic is associated with challenges for obtaining food, housing, income, and medication, which may lead to a rise in drug non-compliance and negative perceptions among sufferers of psychiatric disorders who are already a stigmatized group (50). In general, people with psychiatric disorders exhibit poor physical health, physical co-morbidities, nutritional deficiencies, and short life expectancy (51–53). All these factors increase vulnerability to COVID-19 (54, 55). In fact, the incidence of COVID-19 is high in patients with psychiatric disorders, especially those with depression and schizophrenia (56, 57). Additionally, having a prior psychiatric diagnosis is associated with high mortality among hospitalized COVID-19 patients (53, 57). On the other hand, cytokine storms in severe COVID-19 are reported to trigger damages in the central nervous system resulting in the development of psychiatric disorders (e.g., post-traumatic stress disorder (PTSD), depression, sleep disorder, etc.) in a considerable proportion of recovering COVID-19 patients (56, 58).

The emotional influence of COVID-19 on vulnerable groups such as people with psychiatric problems needs to be further explored (43), with less known about patients in the Arab world, which comprises 22 countries inhabited by 423 million people (59). To bridge the gap, the current study evaluated psychological distress and COVID-19-related psychological trauma in a sample of Arab patients with psychiatric disorders. We hypothesized that COVID-19-related psychological trauma would predict psychological distress. We also hypothesized that participants' perceptions of COVID-19 (as a worrisome condition, high perception of susceptibility to the disease and less likelihood of getting recovered) and prolonged staying at home would be associated with higher levels of psychological distress and psychological trauma. COVID-19 frequently strikes patients with chronic diseases (e.g., diabetes, hypertension, etc.) (55, 60), and COVID-19-related distress is reported to be high among people with chronic disorders (61). Accordingly, we expected that people with perceived poor physical health and those with co-morbid physical disorders would experience more distress and trauma symptoms. We also proposed that patients working or having a family member working in the healthcare field would experience more trauma and distress. General anxiety and COVID-19 conspiracy beliefs among psychiatric inpatients (major depression and substance abuse) in the UK is significantly associated with COVID-19 countermeasure necessity and compliance such as social distancing and political restrictions (33). In parallel, frequent use of precautionary measures (e.g., handwashing with hydroalcoholic solution and mask wearing regardless of the presence or absence of symptoms) is associated with higher psychological distress in the general public in Spain (62). Therefore, we assumed that patients with higher levels of distress or trauma would use more protective measures than patients with lower levels of distress or trauma.

## Methods

### Study Design, Participants, and Procedure

An online questionnaire administered via Google Forms was distributed through WhatsApp and Twitter groups to 1160 anonymous respondents from Saudi Arabia. All participants who reported an age of 18 years or above and signed a digital informed consent were directed to the questionnaire. Data were collected during the official confinement period in Saudi Arabia over the course of four days between April the second and April the fifth, 2020. For this cross-sectional study, 168 respondents reporting a pre-existing diagnosis of a psychiatric disorder, which is diagnosed by a psychiatrist were recruited. The study plan was approved by the Institutional Review Board of Al Qassim University (No. 19-08-01).

### Study Instruments

The structured questionnaire used in this study consisted of several parts. Part 1 comprised sociodemographic and clinical data such as age, income, education, employment, marital status, family size, type of household, working or having a family member working in the medical field, having a chronic physical disorder, health changes in the past 14 days (experiencing symptoms of fever, nasal congestion, muscle ache, etc.), visiting doctor/hospital or being admitted to the hospital during the past 14 days, direct and indirect contact with suspected or confirmed COVID-19 patients, contact with surfaces/tools contaminated with the virus causing COVID-19, being screened for, quarantined, or diagnosed with COVID-19.

Part 2 comprised perceptions and attitudes toward COVID-19—perceived physical health was assessed by one question “rate your physical health status on a scale from 1 = very bad to 5 = very good”; perceived vulnerability to COVID-19 was assessed by one question “rate your perceived vulnerability to COVID-19 on a scale from 1 = very unvenerable to 5 = very vulnerable”; perceived possibility of recovery if they contract COVID-19 was assessed by one question “rate the possibility of your recovery from COVID-19 if you get infected on a scale from 1 = very low to 5 = very high”; confidence in COVID-19 diagnostic methods was assessed by one question “rate your confidence in the methods used to diagnose COVID-19 on a scale from 1 = very unconfident to 5 = very confident”; perception of COVID-19 as a worrisome condition was assessed by one question “rate your agreement with the statement “there is extreme unnecessary worry concerning COVID-19 on a scale from 1 = strongly disagree to 5 = strongly agree”.

Part 3 inquired about protective measures used by the respondents against COVID-19 such as wearing mask, keeping a one-meter distance, avoiding sharing eating utensils at household, and hand washing, along with the duration of being in self-isolation/stay-at-home.

Part 4 inquired about patients' information on COVID-19-related death rates, and the development of drugs or vaccines for COVID-19, their sources of information, and their satisfaction with the available information “How satisfied are you with the information available on COVID-19?”, 1 = very unsatisfied to 5 = very satisfied.

Part 5 comprised the Arabic version of the Depression Anxiety Stress 21 (DASS-21) (63). The scale comprises 21 items in three subscales, each comprising 7 items, which measure symptoms of depression, anxiety, and stress over the past seven days. Item responses are rated on a 4-point scale that ranges from 0 (did not apply to me at all) to 3 (applied to me very much or most of the time). The overall score of the scale reflects psychological distress. The Arabic DASS-21 has been validated previously (64–66), and its reliability in the current sample is excellent (α = 0.96) (63). In our analysis, we used the total score of the DASS-21 not of the subscales. This is because psychometric evaluations of the Arabic DASS-21 indicate its usefulness as a unidimensional measure of distress rather than being a distinct measure of depression, anxiety, and stress (64, 65).

Part 6 comprised the validated Arabic version of the Impact of Event Scale-Revised (IES-R) (67). The IES-R comprises 22 items in three subscales, which describe major features (intrusion, avoidance, and hyperarousal) of PTSD relevant to a specific trauma (68): psychological trauma relevant to the COVID-19 outbreak in this study. In this regard, each item on the IES-R has been altered to make the experience it depicts relevant to the COVID-19 outbreak such as thought of COVID-19 when I didn't mean to (item 6), pictures of the COVID-19 pandemic popped into my mind (item 9), tried not to think about COVID-19 (item 11), had sudden waves of strong feelings about COVID-19 (item 16), reminders of COVID-19 induced physical reactions such as sweating and palpitation (item 19), and had dreams about COVID-19 (item 20). The extent of distress induced by traumatic symptoms relevant to COVID-19 are rated on a 5-point equal response intervals (from 0 to 4), with higher scores indicating higher levels of traumatization (69). Internal consistency of the IES-R in the current sample is excellent (α = 0.92).

### Statistical Analysis

Quantitative variables with non-normal distribution were described using the median and interquartile range (IQR: 25–75%). Categorical variables were described using number and percentage. Independent-sample *t*-test and one-way ANOVA were used to describe between group differences in the DASS-21 and IES-R scores. A series of Spearman correlations involving sociodemographic variables and risk factors for psychological distress and psychological trauma (e.g., having family members working in the medical field, perceived vulnerability to COVID-19, etc.) with the DASS-21 and the IES-R were conducted. A structural equation model (SEM) predicting psychological distress and COVID-19-related trauma included variables with significant correlations. To improve model fit, most non-significant predictors/direct paths were trimmed/eliminated from the model, except for those relevant to key predictors (e.g., staying at home, co-morbid physical disorders, and age) because they are relevant to the addressed hypotheses and model fit was already good. Maximum likelihood with a bootstrap involving 2000 random samples was used to obtain 95% bias-corrected confidence interval for all effects (70). Model fit was considered good based on a non-significant chi-square (χ^2^) index, along with comparative fit index (CFI) and Tucker-Lewis index (TLI) >0.95, in addition to root mean square error of approximation (RMSEA) and standardized root-mean-square residual (SRMR) <0.06 (71). The analyses were conducted in SPSS and Amos, and significance was considered at a probability of less than 0.05 in two-tailed tests.

## Results

This study recruited 168 anonymous patients with psychiatric disorders through a web survey in Saudi Arabia during the lockdown period. The sociodemographic characteristics of the participants are described in Table 1. The majority of respondents were females. Forty-five (26.8%) respondents reported having a chronic physical disease (e.g., diabetes, hypertension, etc.). None of the respondents worked in the medical field while 13.7% of the respondents had a family member working in the medical field. Regarding family size, 33.3% of the respondents came from families comprising 3–5 members while 56.5% came from families comprising more than six members; the rest came from families comprising two members or less. As for the type of household, 56.5% of the respondents lived in villas, 17.3% lived in floors on villas while 29.2% lived in apartments. Independent sample *t*-test and one-way ANOVA test (Supplementary Materials) revealed significant differences in psychological distress scores among groups of age, marital status, and employment (*p* = 0.009, 0.007, and 0.004) while psychological trauma scores were significantly different only among education groups (*p* = 0.039).

**Table 1:** Sociodemographic characteristics of the participants.

**Sociodemographic characteristics**	**(*N* = 168) No (%)**
Gender Females Males	119 (70.8) 49 (29.2)
Age (years) 18–30 >31	87 (51.8) 81 (48.2)
Marital status Single Married Divorced/widowed	80 (47.6) 77 (45.8) 11 (6.6)
Education School education University degree	51 (30.4) 117 (69.6)
Employment Employed Unemployed	49 (29.3) 139 (82.7)
Monthly income (Saudi Rial▴) <15000 >=15000	94 (56.0) 74 (44.0)

*▴: One Saudi Rial is equivalent to 0.27 US Dollar or 0.23 Euro*.

GAD and depressive disorder were the most commonly reported psychiatric diagnoses (Table 2). Co-morbidity was recorded. Sleep disorders, obsessive compulsive disorder (OCD), and eating disorders were the mostly noted co-morbid conditions among patients with GAD and depressive disorder. Independent *t*-test revealed that psychological distress scores did not vary between groups of physical disorders or among groups of different psychiatric diagnoses (all *p* values > 0.05, Supplementary Materials). However, patients with depressive and sleep disorders expressed significant differences in COVID-19-related psychological trauma t(160.2) = −3.21, *p* = 0.002 and t(69.5) = 2.41, *p* = 0.019, respectively.

**Table 2:** Descriptive statistics of the clinical characteristics of the participants.

**Clinical characteristics**	**(*N* = 168)**
Diagnosis Anxiety disorders Depression Sleep disorders OCD Eating disorders PTSD Other disorders▴	70 (41.7%) 68 (40.5%) 40 (23.8%) 26 (15.5%) 15 (8.9%) 12 (7.1%) 34 (20.3%)
Having chronic physical disorder Yes No	123 (73.2%) 54 (26.8%)
IES-R MD (Q1-Q3)	30.0 (14.0–43.0)
DASS-21 MD (Q1-Q3)	21.0 (6.0–39.8)

*▴: Other disorders included personality disorders, bipolar disorder, and psychotic disorders, OCD, obsessive compulsive disorder; PTSD, post-traumatic stress disorders; DASS-21, Depression Anxiety Stress Scale-21; IES-R, Impact of Event Scale-Revised; MD, median; Q1, first quartile; Q3, third quartile*.

Direct and indirect exposure to someone suspected to have COVID-19 as well as exposure to surfaces/tools infected with the virus were reported in 1.2% of the respondents while the rest reported that exposure did not happen or did not know if they were exposed or not. As for health changes in the past 14 days, 31.1, 19.8, 17.4, 15.6, and 15.0% of the respondents reported symptoms of headache, muscle ache, dizziness, sore throat, and nasal congestion while 47.3% of the respondents reported not experiencing any symptoms. Of all the respondents, 19.0% visited the hospital or contacted a doctor in the past 14 days, 0.6% were admitted to the hospital, 3.6% were tested for COVID-19, 1.2% were quarantined for COVID-19, and none were diagnosed with COVID-19. COVID-19-related psychological trauma scores were higher in patients experiencing dizziness t(44.5) = −2.53, *p* = 0.015 and lower in patients not experiencing symptoms in the last 14 days t(165.3) = 2.32, *p* = 0.021. Psychological distress scores were significantly higher among patients experiencing sore throat t(31.89) = −2.64, *p* = 0.013 and difficulty breathing t(19.46) = −3.18, *p* = 0.031.

Descriptive statistics of items of the DASS-21 (Supplementary Material) indicate that feeling down-hearted and blue was the most commonly experienced symptom; median (Q1-Q3) = 2.0 (1.0–3.0), followed by being unable to become enthusiastic about anything feeling rather touchy; median (Q1-Q3) = 1.0 (0.0–3.0), and feeling that life was meaningless; median (Q1-Q3) = 1.0 (0.0–2.8). Mouth dryness, breathing difficulty, and trembling (e.g., hand) were the least reported symptoms; median (Q1-Q3) = 0.0 (0.0–1.0) followed by felt close to panic; median (Q1-Q3) = 0.0 (0.0–2.0). The most commonly reported symptoms on the IES-R (Supplementary Material) were avoided letting myself get upset when I thought about it or was reminded of it, thought about it when I did not mean, stayed away from reminders, tried not to think about it, had trouble concentrating, felt watchful and on guard, and tried not to talk about it; median (Q1-Q3) = 2.0 (0.0–3.0).

Table 3 shows that the majority of the respondents perceived their health status as good. However, 58.9% perceived themselves as vulnerable to COVID-19. Most respondents (69%) perceived COVID-19 as a worrisome condition—the mean score of respondents' agreement to the statement “there is extra unnecessary worry about COVID-19” was 2.0 ± 1.4. Scores below 3 on this item indicate disagreement to the statement. A considerable proportion of the participants had high confidence in the available diagnostic measures of COVID-19, and they perceived their possibility of recovery would be high if they contract COVID-19.

**Table 3:** Participants' perceptions of their general health status, COVID-19 diagnostic methods, their vulnerability to COVID-19, the possibility of their recovery if they contract COVID-19, and COVID-19 as a worrisome condition.

**Patients' perceptions**	**(*****N*** **=** **168)**		
	** <3 No (%)**	**3 No (%)**	**>3 No (%)**
General physical health status	14 (8.3)	37 (22.0)	117 (69.7)
Confidence in COVID-19 diagnose methods	7 (4.2)	32 (19.0)	129 (76.8)
Perceived vulnerability to COVID-19	99 (58.9)	51 (30.4)	18 (10.7)
Perceived possibility of personal recovery if you contract COVID-19	18 (10.7)	37 (22.0)	113 (67.3)
There is unnecessary worry concerning COVID-19	116 (69.0)	23 (13.7)	29 (16.3)

Acknowledging the Saudi Ministry of Health as their main source of COVID-19-related information, most patients reported being updated with the latest news on COVID-19 deaths/and number of infected cases as well as the news on drug/and vaccine discovery. No statistically significant differences in trauma and distress scores were noted among those following the latest news on COVID-19-related deaths/infected cases or the development of COVID-19 drugs or vaccines or those using various sources of information on COVID-19 (Supplementary Material).

Only 9.6% of the participants did not use protective measures and wearing a mask was less common. Handwashing, avoiding hand shake, and keeping a one-meter distance were commonly used by most participants (Table 4). There were no significant differences in the scores of psychological trauma and psychological distress among those using different protective measures. Only those who avoided sharing eating utensils at household expressed a statistical significant difference in psychological trauma t(54.6) = −2.18, *p* = 0.034. The scores of psychological trauma and psychological distress significantly varied among those with partial and complete compliance with stay-at-home orders t(127.8) = 2.50, *p* = 0.014 and t(127.2) = 2.21, *p* = 0.029, respectively.

**Table 4:** Participants' sources of COVID-19-related information and their use of protective measures against COVID-19.

**COVID-19-related information and protective measures**	**(*N* = 168) No (%)**
Updated with the news on COVID-19 deaths/infected cases	
Yes No	153 (91.1) 15 (8.9)
Updated with the news on drugs/vaccines for COVID-19	
Yes No	117 (69.6) 51 (30.4)
Sources of information Social Media Local mass Media Ministry of health World Health Organization	69 (20.9) 53 (16.1) 137 (41.5) 71 (21.5)
Satisfaction with the available information on COVID-19 mean (SD)	4.2 (1.0)
Protective measures Wearing mask Washing hands Avoiding handshake Keeping distance for one meter Avoiding sharing eating utensils Doing nothing	30 (18.0) 140 (83.8) 105 (62.9) 82 (49.1) 38 (22.8) 16 (9.6)
Home stay less than 12 hours per day▴ Not going outside at all	107 (63.7) 61 (36.3)

*▴: One participant stayed at home for up to 18 hours per day*.

As shown in Table 5, psychological distress and psychological trauma were strongly correlated. While psychological distress significantly correlated with age, marital status, and employment; psychological trauma correlated only with education among all sociodemographic factors. Monthly income was not correlated with either distress or trauma (*p* > 0.05, Supplementary Material). Both psychological distress and psychological trauma positively correlated with perceived vulnerability to COVID-19 and negatively correlated with perceived health status and perceived possibility of personal recovery. Psychological trauma negatively correlated with home stay and confidence in diagnostic methods of COVID-19. Perceiving COVID-19 as a worrisome condition correlated with psychological trauma (*r* = 0.155, *p* = 0.045) but not with psychological distress (Supplementary Material). Satisfaction with the available information on COVID-19 was negatively correlated with psychological distress and COVID-19-related trauma (*r* = −0.247 and −0.255, *p* values = 0.001). Psychological trauma negatively correlated with lack of use of any protective measures (*r* = −0.187, *p* = 0.015) and positively correlated with not sharing eating utensils at household (*r* = 0.180, *p* = 0.020).

**Table 5:** Correlations among trauma, psychological distress, sociodemographic characteristics, and perception of vulnerability to COVID-19.

**Variables**	**1**	**2**	**3**	**4**	**5**	**6**	**7**	**8**	**9**	**10**	**11**
1. DASS-21	–										
2. IES-R	0.714^**^	–									
3. Age	−0.240^**^	−0.097	–								
4. Sex	−0.054	−0.079	0.122	–							
5. Marital status	0.248^**^	0.081	−0.615^**^	0.014	–						
6. Education	0.064	0.155^*^	−0.146	−0.176	0.077	–					
7. Employment	0.184^*^	0.144	−0.366^**^	−0.271^**^	0.180^*^	0.037	–				
8. Perceived health status	−0.400^**^	−0.348^**^	0.070	−0.061	−0.069	0.005	−0.058	–			
9. Perceived vulnerability to COVID-19	0.297^**^	0.236^**^	0.033	0.052	0.024	−0.143	−0.064	−0.200^**^	–		
10. Confidence in diagnostic methods of COVID-19	−0.150	−0.180^*^	0.070	0.025	−0.064	0.110	−0.100	0.298^**^	−0.163^*^	–	
11. Perceived possibility of personal recovery	−0.208^**^	−0.289^**^	−0.096	−0.037	0.073	−0.082	−0.013	0.396^**^	0.236^**^	−0.180^*^	–
12. Home stay	−0.151	−0.180^*^	0.231^**^	0.367^**^	−0.075	0.053	0.117	0.052	0.097	−0.75	0.097

*,** *: Correlation is significant at the level of 0.05 and 0.01, respectively*.

**Figure 1:**
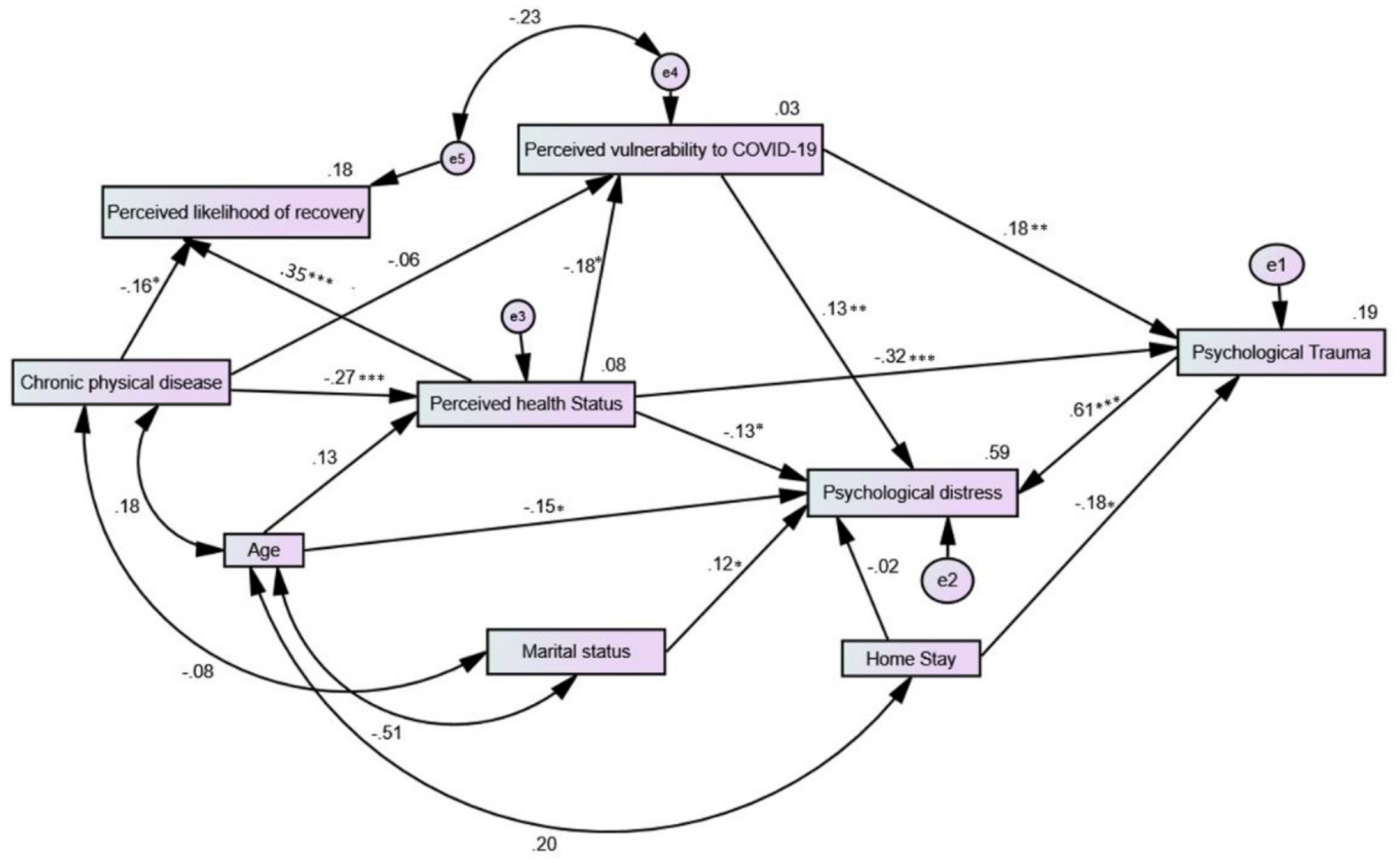
Structural equation path model predicting COVID-19-related psychological trauma and psychological distress in Arab patients with psychiatric disorders.

After trimming most non-significant variables and paths, the SEM path analysis model predicting psychological trauma and psychological distress (Figure 1) had excellent fit on all fit measures (χ^2^ (16) = 13.1, *p* = 0.665, CFI = 1.00, TLI = 1.02, RMSEA = 0.00, SRMR = 0.04). The model accounted for 19.0 and 59.0% of the variances in psychological trauma and psychological distress, respectively. As shown in Figure 1, perceived health status and vulnerability to COVID-19 were strong predictors of COVID-19-related trauma and psychological distress. Age, marital status, and COVID-19-related trauma predicted psychological distress, with the later expressing the strongest effect. Stay-at-home had a significant direct negative effect on COVID-19-related trauma and a significant indirect negative effect on psychological distress mediated by COVID-19-related trauma (β = −0.107, 95% CI: −0.177 to −0.038, *p* = 0.017).

Perceived vulnerability to COVID-19 had a strong indirect effect on psychological distress via COVID-19-related trauma (β = 0.112, 95% CI: 0.039 to 0.184, *p* = 0.009); it also mediated the indirect effect of perceived health status on COVID-19-related trauma (β = −0.033, 95% CI: −0.078 to −0.007, *p* = 0.022). COVID-19-related trauma mediated the indirect effect of perceived health status on psychological distress (β = −0.240, 95% CI: −0.324 to −0.163, *p* = 0.001). Although age had no significant effect on perceived health status, it exerted significant indirect effects via perceived health status on perceived vulnerability to COVID-19 and perceived likelihood of recovery in case of contracting the disease (β = −0.024, 95% CI: −0.065 to −0.004, *p* = 0.047) and (β = 0.046, 95% CI: 0.008 to 0.106, *p* = 0.048), respectively. The indirect effects of age on psychological distress and psychological trauma were marginal (*p* = 0.082 and 0.074, respectively). Having a co-morbid chronic physical disease expressed significant indirect effects on perceived vulnerability to COVID-19, perceived likelihood of recovery in case of contracting the disease, COVID-19-related trauma, and psychological distress via perceived health status (β = 0.050, 95% CI: 0.014 to 0.108, *p* = 0.016), (β = −0.096, 95% CI: −0.172 to −0.049, *p* = 0.000), (β = 0.085, 95% CI: 0.027 to 0.151, *p* = 0.010) and (β = 0.086, 95% CI: 0.022 to 0.150, *p* = 0.018), respectively.

## Discussion

To our knowledge, this is the first study to examine COVID-19-related psychological trauma and psychological distress among Arab patients with psychiatric disorders. COVID-19-related psychological trauma was evident, especially among patients with depression and sleep disorders, and it was a strong predictor of distress. Feeling down-hearted and blue, a depressive symptom, was the most reported distress symptom. Psychological distress was common among patients who were young, unemployed, and single. Staying at home was protective against COVID-19-related psychological trauma and psychological distress. Most participants perceived COVID-19 as a worrisome condition, and those with high perceived poor health status, high perceived vulnerability to COVID-19, and low perceived chance of recovery in case they contract the disease were more likely to exhibit high psychological distress scores.

Although no statistically significant differences in trauma and distress scores were noted between genders (Supplementary Materials), age was a significant negative predictor of psychological distress in our sample, which is consistent with several studies reporting higher distress among youth during the pandemic (3, 9, 37, 72). Age is an important factor that is closely linked to several other interrelated variables (e.g., education, marital status, employment, health status, loneliness, etc.) (70). For example, age was negatively correlated with marital status and employment, which were both positively correlated with COVID-19-related trauma (Table 5). As noted above, age exerted an indirect negative effect on perceived vulnerability to COVID-19 and an indirect positive effect on perceived likelihood of recovery should the patients contract COVID-19. Age was also negatively associated with obtaining COVID-19-related information from the website of the WHO and the Ministry of Health (Supplementary Material). In fact, age along with marital status, educational level, and professional status are reported to affect resilience scores among the general public in several countries during COVID-19, with age expressing the strongest effect among all sociodemographic variables (37). Thus, interventions designed to mitigate COVID-19-related trauma may consider young age as a key effector, especially when it is associated with unemployment, low education, and single marital status.

Contrary to expectations and reports associating high COVID-19 related distress with chronic non-infectious diseases (61), having a chronic physical disorder was not directly associated with COVID-19-related distress or trauma. This may be attributed to the fact that many patients with chronic disorders may enjoy good health, especially when they stick to a healthy lifestyle (adequate exercise, diet, and sleep) (73). This logic may be true given that having a chronic physical disorder negatively predicted perceived health status and exerted indirect effects through that variable on psychological trauma and COVID-19-related distress as well as perceived vulnerability to COVID-19. In line, high levels of psychological distress are reported to prevail when physical disorders are associated with poor health status and low wellbeing such as during periods of active disease (52, 74). In addition to its mediating effect, perceived health status was also a direct predictor of both psychological distress and COVID-19-related trauma. Consistent with our findings, Chinese psychiatric patients with poor physical health expressed more depressive, anxiety, and stress symptoms (44). Likewise, a systematic review pinpoints perceived poor physical health as a predictor of distress among the general public, healthcare providers, and COVID-19 patients (43). Overall, patients with physical co-morbidities, especially those with perceived poor physical health, may be at high risk for COVID-19-related trauma and distress.

Among different psychiatric diagnoses, COVID-19-related trauma symptoms were significantly higher among patients diagnosed with depressive disorder and sleep disorders, which were also comorbid with one another. This finding is consistent with those of an Italian study reporting an association between low sleep quality and high distress in the general public exhibiting COVID-19-related PTSD (72). In fact, a meta-analysis involving cross-trait meta-analysis and Mendelian randomization analysis reports 29 loci shared between PTSD and major depressive disorder, along with a causal effect of genetically determined depressive phenotypes on PTSD. The authors concluded that PTSD, from a genetic point-of-view, is likely to be a subtype of depressive disorders (75). Taken together, depressed patients, particularly those with symptoms of dysfunctional sleep would require special immuno-psychiatric attention in order to prevent the development of COVID-19-related trauma.

Staying at home is reported to contribute to loneliness, decreased social support, and dysphoric mood (23, 24, 36). Contrary to our expectations, prolonged stay-at-home was protective against psychological trauma and distress. This could be related to alleviation of COVID-19 phobia secondary to reduction of direct contact with others (e.g., at work, supermarkets, etc.). In this context, young Italian people who worked outside their domicile during COVID-19 strict lockdown are reported to exhibit higher levels of anxiety and stress than the general public (3). It is also possible that trauma and distress symptoms were low in those with complete compliance with stay-at-home orders due to family interactions and social connectedness associated with large family size—predominantly, more than half the respondents came from families comprising more than 6 members. In support of this view, living with others or in a rural area, having greater social support and more close friends are documented protective factors against loneliness during COVID-19 in the UK (36). In line, perceived social support is reported to moderate the relationship between loneliness and anxiety during COVID-19 in China (76). Longitudinal data indicate that adolescents adhering to stay-at-home orders who feel socially connected are less prone to depression/anxiety, COVID-19 worries whereas those with online learning difficulties, increased conflict with parents, and COVID-19 worries experience an increase in mental health problems during the COVID-19 lockdown (16). On the other hand, data from Canada show that the presence of children under the age of 18 in the household is associated with increased alcohol use, suicidal ideation, parent conflicts with children, domestic violence, worsening of children's mental health as well as more frequent positive interactions with their children and feelings of closeness due to the pandemic (21).

Crowdedness during the confinement period may contribute to distress; however, the perception of human sounds is reported to be context-specific (22). In this study, family size was positively associated with the type of household (*r* = 0.359, *p* < 0.01), with the majority of the respondents living in villas or in a floor on a villa. Thus, the housing conditions would provide plenty of space and privacy. In line, compared with house dwellers, apartment dwellers experience more exposure to mechanical sounds, which is associated with lower self-reported health and lower restorative quality of the home (feeling away) during the lockdown (22).

Although none of the respondents worked in the medical field, some patients had a family member working in the medical field. However, those patients expressed no variation in COVID-19-related trauma or distress scores, which is contradictory to what is reported in the literature (3). This finding would be interpreted within the context of data collection, which took place during the beginning of the confinement period where the number of patients infected with COVID-19 in the entire Saudi Arabia was around 1000. Thus, it is possible that family members working in the medical field may had less contact with COVID-19 patients, entailing less vicarious trauma (4).

### Strength, Implications, and Limitations

This study is the first to describe the psychological impact of COVID-19 and its correlates among Arab patients with psychiatric disorders. It examined psychological distress: non-specific negative emotions of combined feelings of anxiety and depression, which are closely associated with mental disorders (77). This is because the DASS-21 is not a diagnostic measure, and it primarily captures psychological distress rather than discrete symptoms of depression or anxiety (78). In line, a meta-analysis states that the reported incidence of depression and anxiety during the pandemic as assessed by various specific diagnostic measures (e.g., Generalized Anxiety Disorder, Hamilton Depression Scale, etc.) is highly heterogenous (79).

The findings identified some of the key risk factors of mental health consequences of COVID-19, which may inform immuno-psychiatric and resilience promoting efforts toward patients with psychiatric disorders, who represent one of the most vulnerable groups to COVID-19 and its adverse effects. The results highlight the importance of screening (e.g., online, on the phone) patients with psychiatric disorders for COVID-19-related trauma as well as symptoms of distress in order to mitigate mental health risks among those patients. Vulnerable individuals who may need special support are mainly those who are young, single, unmarried, with physical comorbidities, poor perceived physical health, and high perceived vulnerability to COVID-19. Patients diagnosed with major depression and sleep disorders are particularly vulnerable to COVID-19 trauma.

This study also has a number of limitations, which may limit the generalizability of the findings: cross-sectional design, selection bias (by recruiting only educated patients who use social media from a single Arab country), social desirability bias (self-reported data), and recall bias. Psychiatric diagnoses were self-reported, even though they were indicated to be performed by psychiatrists. Because of noted psychiatric comorbidities, it was not possible to investigate the contribution of the main psychiatric diagnosis to COVID-related distress and trauma in SEM. However, collecting data through an online survey was the only convenient way because face-to-face contacts were strictly forbidden during the confinement period. It is worth mentioning that data collection took place early during the pandemic while research signifies a temporary increase in mental symptomatology at the initial periods of the pandemic followed by a drop by mid-2020 to the levels reported before the pandemic (35). In addition, the pre-COVID-19 level of psychological distress in the current sample has not been assessed, which makes us unable to affirm that distress estimated is purely attributed to the pandemic. Therefore, the results must be interpreted with caution. Meanwhile, the pandemic is ongoing and the need to ensure prompt provision of adequate healthcare to acute psychiatric patients remains immense.

## Conclusion

COVID-19-free patients with psychiatric disorders endorse COVID-19-psychological trauma, and subsequently experience psychological distress. Experiencing symptoms of dizziness, sore throat, and difficult breathing was associated with higher COVID-19-related trauma and distress. Patients were up to date with the latest information about COVID-19 mortality and treatment, and the ministry of health was the main source of information in addition to the WHO and social media. Satisfaction with information available about COVID-19 did not correlate with distress or trauma. Patients largely complied with protective measures, and trauma symptoms were higher among those not sharing their eating utensils at household. Sociodemographic variables (age, marital status, and employment), perceived health status, and beliefs about risk of infection and chances of personal recovery significantly predicted distress and trauma. Staying at home was protective against COVID-19 trauma and emotional reactions.

To prevent mental health consequences, the findings suggest that more research attention should be directed toward fostering adaptive coping among young, unemployed, and single patients, especially those with depression and sleep disorders as well as those with physical disorders who perceive their physical health as poor or perceive themselves more vulnerable to COVID-19. Research is needed to investigate whether psychological distress in Arab psychiatric patients is associated with COVID-19-related conspiracy theories as well as burdensome consequences of the outbreak such as difficulties with access to healthcare services as well as availability of job/income, food, support system, etc. Longitudinal investigations are required to inform whether the emotional reaction of psychiatric patients changes over the course of the pandemic.

## Data Availability Statement

The datasets presented in this study can be found in Mendeley repository at: https://data.mendeley.com/datasets/8k3vmfxpd3/draft?a=67415321-61f7-4920-bd2a-749b365ff6fb (access on 27 September 2021).

## Ethics Statement

The studies involving human participants were reviewed and approved by Institutional Review Board of Al Qassim University (No. 19-08-01). The patients/participants provided their written informed consent to participate in this study.

## Author Contributions

AMA, AAA, and AOH conceptualized the study and designed the methodology. AAA collected the data. ESAE and SMT cleaned the data. AMA and AOH analyzed and interpreted the data and edited and revised the final draft. AMA, ESAE, SMT, AAA, and AOH wrote the initial draft of the manuscript. All authors have critically revised and approved the final draft of the manuscript.

## supplementarymaterial

The Supplementary Material containing relevant analyses that are not reported in this article can be found online at: https://www.frontiersin.org/articles/10.3389/fpubh.2021.799812/full#supplementary-material

## Conflict of Interest

The authors declare that the research was conducted in the absence of any commercial or financial relationships that could be construed as a potential conflict of interest.

## Publisher's Note

All claims expressed in this article are solely those of the authors and do not necessarily represent those of their affiliated organizations, or those of the publisher, the editors and the reviewers. Any product that may be evaluated in this article, or claim that may be made by its manufacturer, is not guaranteed or endorsed by the publisher.
